# Investigation of an Epidemic of Malaria in a Military Station

**DOI:** 10.4103/0970-0218.40882

**Published:** 2008-04

**Authors:** PMP Singh, SK Handa, Rajvir Bhalwar, AG Mahendraker, A Banerjee, DK Mandal

**Affiliations:** DADH, HQ 21 Mountain Divisions, C/O 99 APO, India; 1 DDMS MB Area, India; 2 Department OF PSM, AFMC, Pune - 411 040, India; 3 ADH and SR ADV (PSM), 4 CORPS, C/O 99 APO, India; 4 Department of PSM, DY Patil Medical College, Pimpri, Pune, India; 5 Section Hospital, IMA, Dehradun, India

## Introduction

At present, about 100 countries are considered malarious, almost half of which are in sub-Saharan Africa. Urban and periurban malaria are on the increase in South Asia and in many areas of Africa. Unfavourable ecological changes have greatly contributed to malaria epidemics.(1)

## Materials and Methods

Relevant epidemiological history was taken from all the 40 cases on an epidemiological case sheet. Case sheets and laboratory reports were pursued for abnormally high incidence of other mosquito-borne diseases besides malaria. Search for more cases was made by visiting private practitioners and government civil hospital of the city. No additional cases were found by medical survey other than those who were already reported sick. Meteorological data was also noted. Surveillance data obtained from all the above sources and cases was analysed.

In order to control the outbreak the following preventive measures were taken immediately:

- Health education of all troops and families on prevention of malaria was carried out. Early detection of cases/illness was ensured through education.
- All potential mosquito breeding spots were identified and eliminated immediately by intensifying antilarval measures.
- Personal protective measures were strictly enforced.
- Fogging activities were intensified to reduce mosquito density using suitably modified vehicle.
- Larvivorous fishes were procured and used in the static fire-fighting tanks to control mosquito breeding.
- Joint civil military action plan was formulated and launched after holding several meetings with the Medical Superintendent, Rural Hospital and CEO Municipal Corporation.

## Results

The population of the military station was approximately 2200 during the period of the epidemic. A total of 40 cases of malaria occurred in the station between 23 July 2003 and 11 October 2003, thus giving an overall attack of 1.8%. Between 1999 and 2002, only 5, 5, 7 and 8 cases of malaria occurred, respectively, during the corresponding months of July to October.

All cases presented with intermittent fever with chills and rigors, and 37 (92.5%) cases had splenomegaly on clinical examination.

Out of 40, 34 (85%) cases were hospitalized for 10-15 days. Two (5%) cases were hospitalized for more than 15 days. The remaining four (10%) cases were discharged after 7-10 days of hospitalization without any complications. All the cases responded to chloroquine, except one death, which was a case of mixed infection and reported late for treatment. Primaquine radical treatment was also administered to the cases of *Plasmodium vivax* malaria to prevent relapse.

Out of 40 cases, 33(82.5%) were Plasmodium vivax malaria, 6 (15%) were *Plasmodium falciparum* malaria and 1 (2.5%) was mixed infection.

Out of 40, 31 (77.5%) were amongst troops and 9 (22.5%) cases were among families. There was no case reported in officers or officer's families. Nineteen (47.5%) cases were in the age group of 40-50 years, and 12 (30%) were in the age group of 25 to 35 years. All the nine (22.5%) cases from families were in the age group of 25-30 years.

Fifteen (37.50%) cases occurred amongst guards who were deployed at various posts in the area; 16 (40%) were amongst troops residing in different areas of the station, which were in close vicinity of each other, and 9 (22.5%) cases were among families residing in family quarters. The clustering of cases in time and space confirmed the existence of an epidemic.

The first case of malaria occurred on 23 July 2003. This was followed by a gradual rise in the number of cases. The highest number of cases in a single month, viz 13 (32.5%) cases, occurred in September 2003. The last case occurred on 11 October 2003.

A few important environmental factors that favoured the occurrence of the epidemic are discussed below.

- The terrain in the station is uneven, which leads to water logging and creates pools of stagnant water. There was 1107.6 mm of rainfall in the year 2003 as against 754.2, 586.2 and 602 mm during the years 2000, 2001 and 2002, respectively. The temperature in the station during the monsoon season also remains between 22°C and 30°C, which is very conducive for breeding of mosquitoes.
- The military station is located on the bank of an irrigation channel that was under construction. Some amount of mosquito breeding was also observed in 110 static water tanks, which were located inside the military station and were used for fire fighting.
- Vast area of the station hampers adequate antilarval measures where guard posts are located. Similar increased incidence of malaria was also found in the civil population of the station as was evident from local newspaper reports, local TV channels and liaison with Medical Superintendent, Rural Hospital and CEO Municipal Corporation.

## Discussion

Epidemics of malaria have been occurring in all parts of the world. In a study carried out in 2004, overall 553 clinical cases of malaria were reported from 1981 to 2002, with some fluctuation over time but it gradually increased. In these 553 cases, 562 microscopy diagnoses were made with *Plasmodium falciparum* infection contributing to 295 (52.49%) of the diagnoses. Nine (2%) clinical cases were mixed infections, involving *Plasmodium falciparum* with either *Plasmodium malariae* or *Plasmodium vivax*.(2) The findings of this study differ from the present study in that *Plasmodium falciparum* infection rate was six (15%) and mixed infection was one (2.5%) in the present study.

Two ‘epidemics’ were reported at the Tropical Diseases Centre from 1986 to 1990 and from 1999 through 2000. A review of the Federal Health Canada databases for the incidence of malaria in Canada from 1990 through 2002 documents a range from 364 to 1029 cases per year, with an average of 538 cases per year during the period (or an average of ≈1.8 cases per 100,000 populations per year).(2) The incidence in the present study of 1.8% is far higher than this.

The Kilgoris, Kisii and Tabaka hospitals reported 171,312 admissions due to malaria over a total of 54 admission years. The Kilgoris, Kisii and Tabaka hospitals managed an average of 2243, 9191 and 3929 malaria admissions per year, respectively, for the duration over which records were available. The long-term data used in this analysis indicate that clinical cases of malaria occur every month at each hospital; acute seasonal peaks occur in June and July.

On average, one-third of the total annual child malaria admissions were concentrated in these 2 months (35%, 32% and 27% for Kilgoris, Kisii and Tabaka, respectively).(3) The findings of this study differ slightly from the present study as regard the peak incidence, which occurred in September. In high-altitude zones of western Kenya, clinical malaria has an acute seasonal distribution, and is a substantial public health problem every year.(3)

The present study has its limitations in the sense that vector species identification of the mosquito species was not carried out due to limited resources available in the station.

Demanding intensive investment in early detection, warning and forecasting systems and frequent complex, emergency responses by government or nongovernmental organizations may not be the most appropriate and cost-effective use of limited resources. Investment in sustainable approaches to vector control (spraying households with residual insecticide), promoting individual protection (insecticide-treated bed nets) and effective case management are perhaps more likely to achieve long-term reductions.
